# Development and Study of a New Silane Based Polyurethane Hybrid Flexible Adhesive—Part 1: Mechanical Characterization

**DOI:** 10.3390/ma16237299

**Published:** 2023-11-23

**Authors:** Vasco C. M. B. Rodrigues, Eduardo A. S. Marques, Ricardo J. C. Carbas, Michael Youngberg, Anne Dussaud, Reza Beygi, Lucas F. M. Da Silva

**Affiliations:** 1 Institute of Science and Innovation in Mechanical and Industrial Engineering (INEGI), Rua Dr. Roberto Frias, 4200-465 Porto, Portugal; vbrodrigues@inegi.up.pt (V.C.M.B.R.); rcarbas@inegi.up.pt (R.J.C.C.); 2 Department of Mechanical Engineering, Faculty of Engineering, University of Porto, 4200-465 Porto, Portugal; emarques@fe.up.pt (E.A.S.M.); lucas@fe.up.pt (L.F.M.D.S.); 3 Momentive Performance Materials Inc., 769 Old Saw Mill River Road, Tarrytown, NY 10591, USA; michael.youngberg@momentive.com (M.Y.); anne.dussaud@momentive.com (A.D.)

**Keywords:** SPUR, silylated polyurethane, flexible adhesive, adhesive characterization

## Abstract

The need for more sustainable adhesive formulations has led to the use of silane-based adhesives in different industrial sectors, such as the automotive industry. In this work, the mechanical properties of a dual cure two-component prototype adhesive which combined silylated polyurethane resin (SPUR) with standard epoxy resin was characterized under quasi-static conditions. The characterization process consisted of tensile bulk testing, to determine the Young’s modulus, the tensile strength and the tensile strain to failure. The shear stiffness and shear strength were measured by performing a thick adherend shear test. The in-plane strain field was obtained using a digital image correlation method. Double-cantilever beam and mixed-mode tests were performed to assess the fracture toughness under pure modes. The prototype adhesive showed promising but lower properties compared to commercial solutions. Furthermore, the adhesive was modified via the addition of three different resin modifier additives and characterized via measuring the shear and tensile properties, but no enhancements were found. Finally, the adhesive was formulated with three different SPUR viscosities. The critical energy release rate analysis showed an optimum value for the medium viscosity SPUR adhesive.

## 1. Introduction

The increased availability of high-performance adhesives has revolutionized structural design procedures. In specific applications, riveting and fastening processes can be substituted by adhesive bonding or even combined into a hybrid joint. Flexible adhesives also play an important role in the automotive industry, acting as sealants, structural or semi-structural adhesives to achieve a lightweight and eco-friendly design [1,2,3,4,5]. The analysis of the hybrid bond between dissimilar materials [6,7,8,9], as well as its durability [10,11,12], has been developed. Favourable stress distribution and damping properties have made semi-structural adhesives good candidates for use in many applications [1]. More specifically, the polyurethane adhesive family is widely known for its mechanical flexibility, which is useful for bonding composite substrates, lowering stress peaks and leading to a lower risk of failure due to delamination [2,13,14,15]. These adhesives possess a low elastic modulus and relatively low tensile strength, accompanied by a much greater elongation at failure [16,17]. The high strain levels supported by these adhesives are beneficial for their gasket sealing properties [18,19], exceptional damping [20,21] and good impact resistance [22].

Despite the mechanical advantages, common silicone and polyurethane-based adhesives retain a certain level of toxicity [23,24,25,26]. Even when the mechanical performance and cost are satisfactory, environmental concerns are important to take into account necessitating a thorough product life cycle analysis. Silane terminated polymers (STPs) have been developed [27,28,29], to provide flexible resins which contain no isocyanate and have typically a lower toxicity level than their polyurethane counterparts. The two main classes of silane terminated polymers are the silyl terminated polyethers and the silylated polyurethanes. Typical silylated polyurethane resins are obtained via the chain extension of polyols using isocyanate chain extenders and end-capping using silanes (aminosilanes or isocyanate silanes).

The adhesive under study in this work is a two-component dual cure system which combines a moisture cure silylated polyurethane resin (SPUR) base and an epoxy resin base, which undergoes hardening during curing. Blends of silyl-terminated polyethers (STP) with epoxy resins have been characterized by Devroey and Homma [30], using dynamic mechanical analysis, lap shear tests, metal-to-metal T-peel tests and transmission electron microscopy (TEM). Their work showed that for the best adhesive performance, under a specific ratio of STP/epoxy resin, the epoxy resin formed discrete domains within the elastic STP crosslinked matrix. The reinforcement by the epoxy resin led to an 8 to 10 times increase in the peel strength compared to the T-peel strength of the adhesive without epoxy resin. Epoxy/STP blend-based adhesives have been further investigated by Bitenieks et al. [31]. Their types of characterization and formulations were mostly focused on the bonding applications for the construction market. The SPUR/Epoxy blends still remain poorly studied, and there is still a need for a detailed mechanical characterization of this type of dual cured adhesives for the transportation applications.

In the present study, the silylated polyurethane resins were obtained via the chain extension of polyols using isocyanate chain extenders and end-capping with isocyanate trimethoxy silane. The second component of the adhesive contained epoxy resin, water and a catalyst. In addition to the main mechanical characterization, the adhesive composition was further modified by the addition of three different additives to analyse its influence in both shear and tensile properties. Lastly, the critical energy release rate value obtained for three different SPUR viscosity levels (low, medium and high) was studied to perceive whether the viscosity level influences the mode I value and how. Specifications for such additives and viscosity levels were not provided due to the adhesive prototype level and development state.

## 2. Materials

The two-component dual cure adhesives and the resin modifier additives were provided by Momentive Performance Materials Inc.^®^ (Niskayuna, NY, USA). The first part of the adhesive contained the SPUR resin, the amine hardeners and the fillers. The second part contained the epoxy resin, water and the catalyst. The resin additive modifiers were added to the first part of the base adhesive before combining both parts.

The base formula of the adhesive was prepared with three SPUR resins having different molecular weights: a low viscosity SPUR referred to as “L”, a medium viscosity SPUR referred to as “K” and a high viscosity SPUR referred to as “J”. The adhesives analysed via mechanical characterization are listed below:

- Three different resin modifier additives;
- Adhesive prepared with three different molecular weight SPU resins.

## 3. Methodology

All tests were performed under quasi-static conditions at room temperature (20 °C) in an INSTRON^®^ 3832 (Norwood, MA, USA) quasi-static machine with a load cell of 30 [kN]. For DIC measurements, a digital camera (Canon EOS M5) was used. The camera lens was a Canon^®^ EF-M 18–55 mm F/3.5–5.6. The DIC measurement was performed with GOM Correlate^®^ (2019) software.

### 3.1. Tensile Test

The bulk tests were performed to obtain the adhesive tensile properties: Young’s modulus, tensile strength and tensile strain to failure [32]. Bulk specimens were obtained by curing the adhesive into a mould under hydrostatic pressure to form an adhesive sheet which was machined into dog-bone specimens [32,33]. All specimens were tested at a constant rate of 1 [mm/min].

#### Bulk Sheet Manufacture

**Figure 1:**
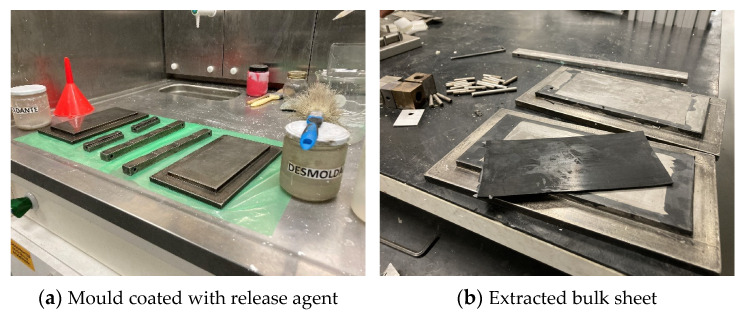
Details of procedures during the bulk specimen fabrication.

For polyurethane adhesives, several authors [32] have adopted the injection moulding fabrication. However, due to the existence of epoxy addition, the current adhesive could be machined without having any ripping-out issues. The mould used for this purpose was described in [33]. The thickness of the bulk sheet was set using a 2 [mm] thick silicone rubber frame, preventing the adhesive from flowing away from the mould cavity, while creating a hydrostatic pressure that prevented bubble and void formation. Before use, the mould was degreased with acetone (Figure 1a) and coated with a release agent.

The adhesive was poured into the assembled mould and placed in a hot press, where the pressure was set to 30 [bar] and kept at 50 °C for 24 h.

**Figure 2:**
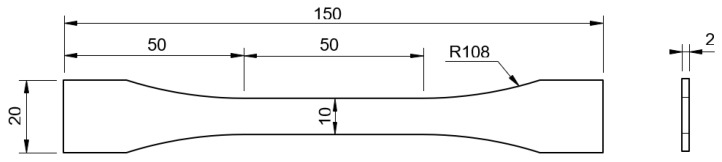
Dog-bone specimen dimensions [mm].

The overall dimensions of the bulk sheet were 155 × 70 [mm], with a thickness of 2 [mm]. The sheet, shown in Figure 1b, was longitudinally cut into three separate parts, which were then machined in a HAAS VF2 Mini. This process guaranteed that the bulk specimen dimensions followed the French NF T 76-142 standard (Figure 2) [34]. To ensure stiffness during machining, small glass fibre tabs were inserted in-between the three stacked adhesive sheets.

### 3.2. Thick Adherend Shear Test

TAST was conducted to determine the shear properties of the adhesive (shear strength and shear modulus). The steel substrates of each specimen are thick to minimize peel stresses. Single lap joint testing has often been used to assess the adhesive properties under shear, but it exposed the adhesive to a complex stress state caused by peel stresses, as a result of the thin substrate. TAST specimens avoided these complex stress configurations and allowed a more accurate extraction of shear strength and shear modulus data [19,20].

All specimens were tested at a crosshead rate of 1 [mm/min].

#### Specimen Manufacture

**Figure 3:**
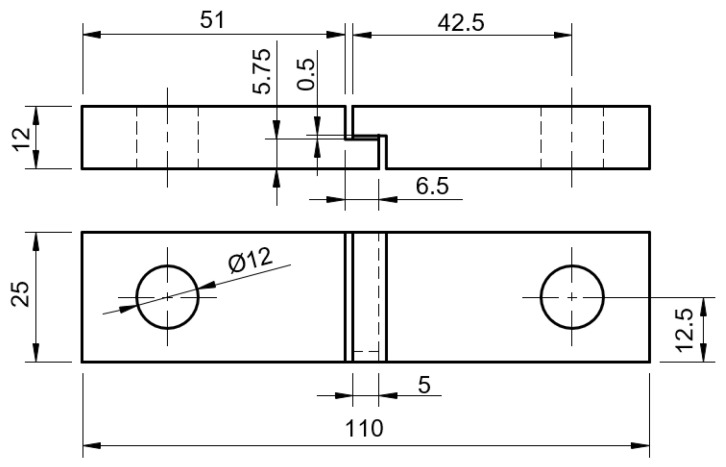
TAST specimen dimensions [mm].

A special mould was used for manufacturing six TAST specimens, each possessing an adhesive thickness of approximately 5 [mm] and 1.5 [mm] thick shim tabs. The shims controlled the adhesive overlap and avoided tensile stresses during testing. The adhesive thickness was measured for each specimen pair using callipers with a resolution of 0.05 [mm]. Figure 3 displays the dimensions of the specimens used following ISO 11003-2 standards [35] [mm]. 

**Figure 4:**
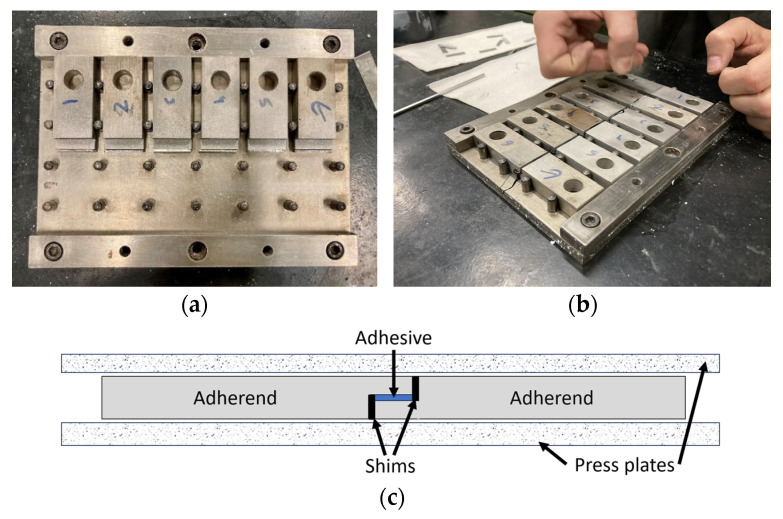
TAST mould preparation: (**a**) mould with guiding pins; (**b**) specimen assembly; (**c**) shims assemblage.

The mould base was cleaned with acetone prior to the release agent application. The bottom TAST adherends were placed as shown in Figure 4c. The adhesive was applied to the bottom adherents following the lower shims and the top part assembly as shown in Figure 4c. Hereafter, 1.5-mm-thick shims were placed in each specimen, to prevent the adhesive from reaching any unwanted surfaces, thereby creating an almost pure shear condition during testing [32]. The top plate was placed over the assembly and the mould was inserted into a thermal chamber with a weight on top, following the same curing conditions as described in the previous section.

After curing, the specimens were carefully withdrawn from the mould, and the excess adhesive was removed using a sharp cutter. A fine sandpaper was used to smooth the side surfaces. This process was carefully performed in order to not create cracks or break the joint, which might happen when removing the shims [19]. Finally, the specimens were prepared for DIC analysis by coating with white matte paint followed by the application of black speckling.

### 3.3. Double Cantilever Beam Test

The DCB specimens were used to perform a fracture mechanics-based analysis of the adhesive behaviour. Adherends made of DIN 40 CrMnMo 8-6-4 steel were used to estimate the critical strain energy release rate, the so-called *G_I__c_* or mode I fracture toughness. From the load versus displacement curve from the testing machine, it was possible to estimate the energy release rate value by monitoring the crack size throughout the assay. All DCB specimens were tested at a constant rate of 0.2 [mm/min].

#### Fracture Specimen Fabrication and Formulation

**Figure 5:**
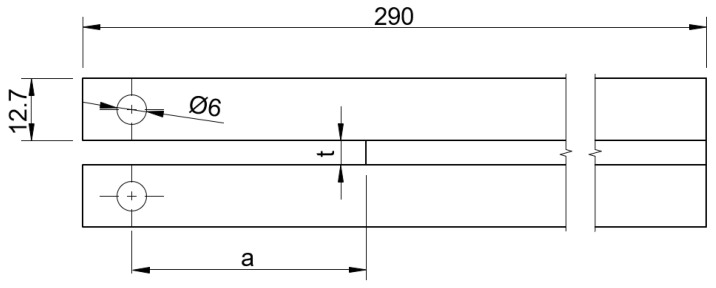
DCB adhesive joint dimensions [mm].

The double cantilever beam adherends used conformed with ISO 25217 standard [36], as shown in Figure 5.

**Figure 6:**
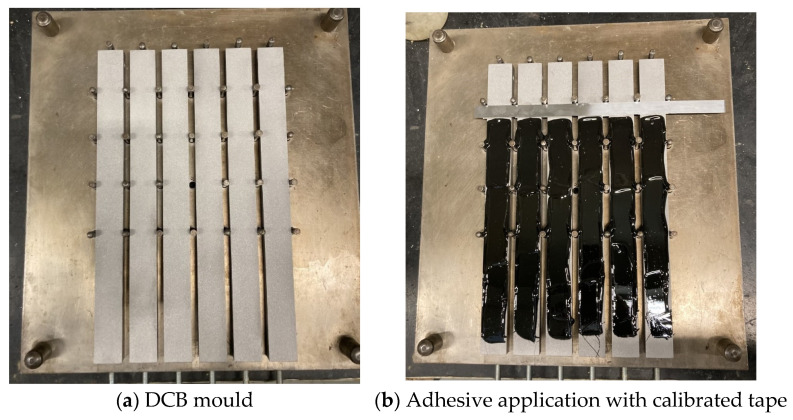
Preparation of DCB specimens.

Specimens were first cleaned with acetone and sandblasted. The bottom substrates were inserted into a clean mould with guiding pins [32], previously coated with the mould release agent (Figure 6a). To obtain an adhesive thickness of 0.20 [mm], a calibrated tape was used on both specimen sides. The crack was moulded using a 0.10 [mm] sharp razor blade with two pieces of 0.05 [mm] calibrated tape attached on both sides. This assembly was used to create an initial crack *a*_0_ of about 45 [mm]. A piece of 0.20 [mm] thick tape was placed at the end of the specimens. These two components were cleaned and coated with a release agent film to facilitate its removal after the curing process. The adhesive was applied on both the bottom (Figure 6b) and top substrates, which were assembled over the top mould part. The mould was placed into a thermal chamber and loaded with weights to prevent voids and ensure that the curing specifications were met.

The critical energy release rate in mode I (*G_I__c_*) was determined using the Compliance Based Beam Method (CBBM) [37], a methodology that based its formulation on an equivalent crack length concept which directly depended on the specimen compliance throughout the test. The R-curve was computed based only on the P-*δ* data provided by the testing machine, without the need for recording the instantaneous crack length, which is difficult to accurately assess. Equation (1) shows the expression of the critical energy release rate *G_I__c_*.

(1) $$G_{I_{c}}=\frac{6P^{2}}{B^{2}t}\left(\frac{2a_{eq}^{2}}{t^{2}E_{f}}+\frac{1}{5G}\right)$$

The geometrical parameters *B* and *t* represent the adherend width and height, respectively, whereas *E_f_* is the corrected flexure modulus, *P* is the instantaneous loading and *a_eq_* is the equivalent crack length, which assesses the real crack tip *a*. A crack correction factor ∆ for the tip deflection and rotation phenomena as well as ∆*a_FPZ_*, another correction, took the fracture process zone effect into consideration [37,38].

(2) $$a_{eq}=a+\Delta +\Delta a_{FPZ}$$

### 3.4. Mixed-Mode Fracture Characterization

The End-Notched Flexure (ENF) test is usually performed [2,32] to determine the fracture toughness in mode II. However, for flexible adhesives such as silicone adhesives, given the large fracture process zone (FPZ), the crack did not propagate at all, or it nucleated after the substrates exceeded the elastic region. ENF tests were performed, but the crack only began to propagate following plastic yielding of the steel substrates, which resulted in an excessively high *G_II__c_* value. Given the inability to test this adhesive under pure mode II conditions, a mixed-mode strategy was chosen to estimate the critical energy release rate by correlating the adhesive fracture envelope.

**Figure 7:**
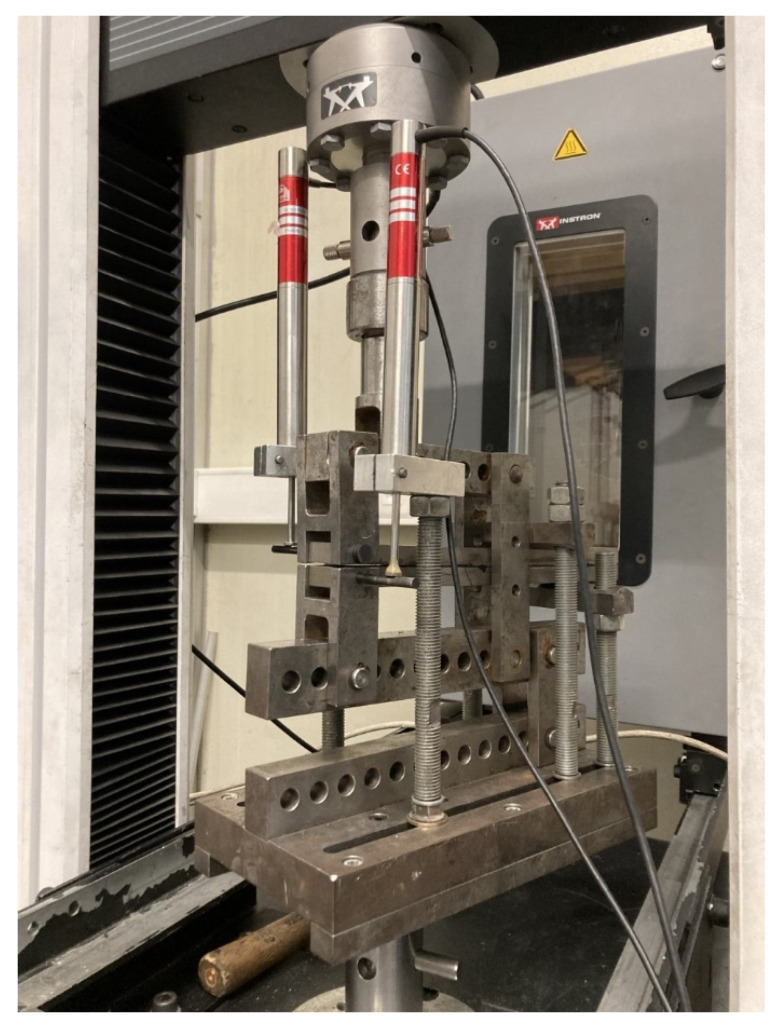
Apparatus for mixed-mode test.

**Figure 8:**
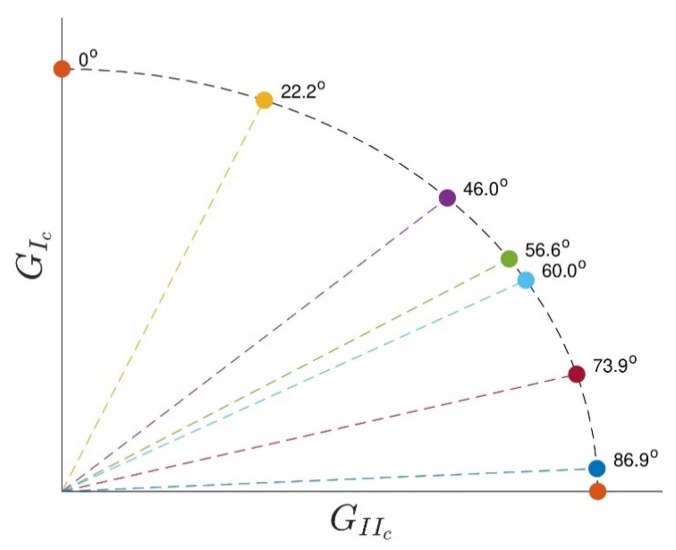
Example of a fracture envelope extrapolation with different phase angles under mixed-mode testing.

The apparatus for the mixed mode, described in [39], allowed the determination of the *P* − *δ* curve for both modes, using only the load versus displacement curve provided by the testing machine and the data from two Linear Variable Differential Transformers (LVDTs), each one measuring the displacement of each substrate (Figure 7). The apparatus was flexible in terms of the mixed-mode ratios it could test. The relative contribution of each mode (I and II) was easily varied by changing the phase angle of the apparatus. This was induced by reassembling the apparatus according to [39]. The relationship between the phase angle *ϕ* and the ratio of the fracture toughness in mode II to fracture toughness in mode I is shown in Figure 8. This method took into account the amount of each mode loading, opening and in-plane shear.

(3) $$\Phi =arctan\left(\sqrt{\frac{G_{II}}{G_{I}}}\right)\;,\mathrm{where}\;\left\{\begin{array}{c} \Phi =0^{{}^{\circ}}\\ 0^{{}^{\circ}}<\Phi <90^{{}^{\circ}}\\ \Phi =90^{{}^{\circ}} \end{array}\begin{array}{c} ,\mathrm{pure}\;\mathrm{mode}\;I\\ ,\mathrm{mixed}\;\mathrm{mode}\\ ,\mathrm{pure}\;\mathrm{mode}\;\mathrm{II} \end{array}\right.$$

**Figure 9:**
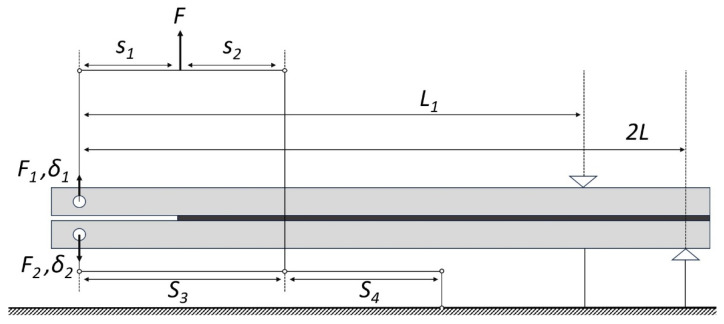
Representation of the specimen in the apparatus for mixed mode.

The procedure used with this apparatus considered the specimens compliance, extension and loading to further estimate an equivalent crack length. This overcame the difficulty of measuring the instantaneous crack size. In the DIC correlation direct methodology, the FPZ is not considered during calculations [40]. Instead, in the indirect approach adopted here, conventional crack tip measurements were replaced by equivalent crack lengths calculated from the compliances of the DCB beams obtained throughout the test. Figure 9 displays the forces to which each DCB specimen was subjected. In the device, six geometric parameters (*s*_1_, *s*_2_, *s*_3_, *s*_4_, *L*_1_, 2*L*) could be adjusted to produce different phase angle values. The estimation of the fracture energy was performed by joining the pure mode I (DCB test) and pure mode II loading cases (ENF test), combining a compliance-based beam methodology (CBBM) [39]. The compliance of the specimen beams for mode I and II, respectively, are expressed as follows:

(4) $$C_{I}=\frac{8a^{3}}{EBh^{3}}+\frac{12a}{5BhG_{13}}$$

(5) $$C_{II}=\frac{3a^{3}+2LL_{1}^{2}}{2EBh^{3}}+\frac{6LL_{1}}{5BhG_{13}\left(2L-L_{1}\right)}$$

where *B* and *h* are geometric parameters, corresponding to the beam width and thickness; *E* and *G*_13_ are the Young’s modulus and flexural modulus of each substrate, respectively. The crack length is represented by *a* and the geometric parameters *L*_1_ and 2*L* are point supporters of the specimen displayed in Figure 9. The equivalent crack length can be calculated following the CBBM for mode I [34] and mode II [41].

Table 1 summarizes the geometrical parameters values for the three different configurations tested [39]. The quasi-static test was performed at a rate of 0.2 [mm/min].

## 4. Results

This section presents a comprehensive analysis of the mechanical behaviour of the 2k SPUR adhesive; the impact of the addition of the three resin modifier additives to the adhesive mixture on tensile and shear properties; and the impact of the SPUR viscosity used in the adhesive mixture on the fracture toughness value.

### 4.1. 2k SPUR Adhesive

#### 4.1.1. Tensile Test

**Figure 10:**
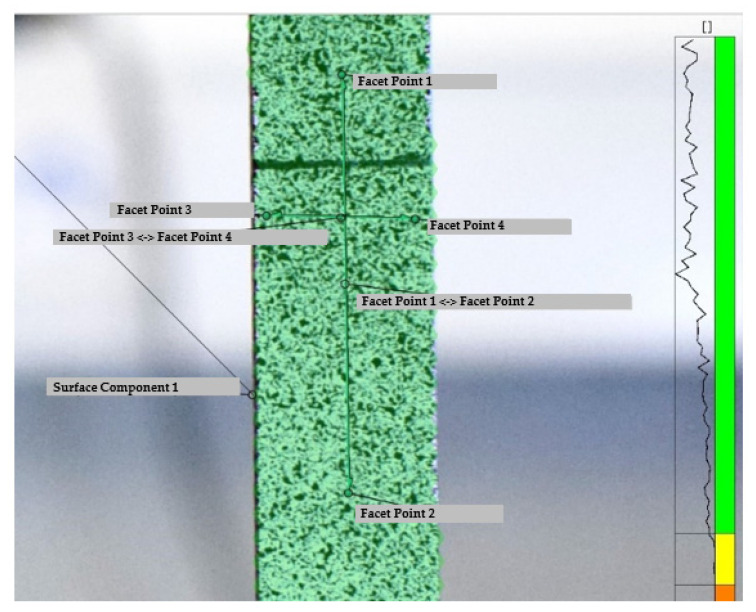
Speckle quality in GOM Correlate^®^ software.

**Figure 11:**
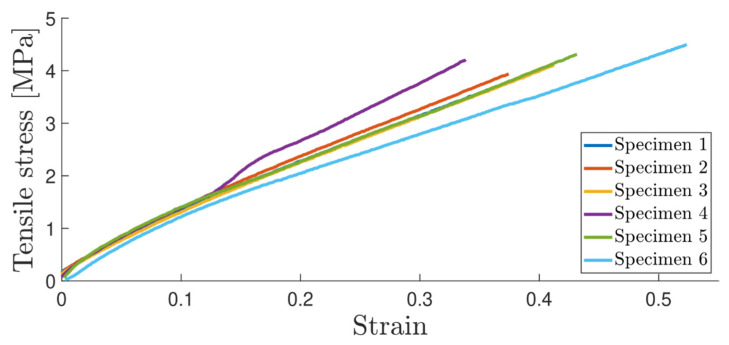
Tensile stress–strain curve.

The surface of the dog-bone specimens was cleaned with acetone and sprayed with a matte white paint prior to speckling with a dark matte paint. Two marks 30 [mm] apart were drawn on the specimen. Six specimens were positioned in pinned fixtures for testing. The strains were computed using DIC software and the stress–strain curve was obtained. Figure 10 illustrates the pictures taken in the DIC software. The stress strain curve is illustrated in Figure 11 for the six specimens tested. Table 2 summarizes the average values as well as the standard deviation for the Young’s modulus, tensile strength and tensile strain to failure.

From these results, it was possible to estimate the Poisson’s ratio *ν* considering a DIC analysis, as illustrated in Figure 10, using the following expression:

(6) $$\nu =-\frac{\varepsilon_{x}}{\varepsilon_{y}}$$

where *ε_x_* and *ε_y_* correspond to the strain field in the *x* and *y* direction, respectively. A Poisson’s ratio value of *ν* = 0.418 ± 0.009 was found for the presented adhesive.

#### 4.1.2. Thick Adherend Shear Test

**Figure 12:**
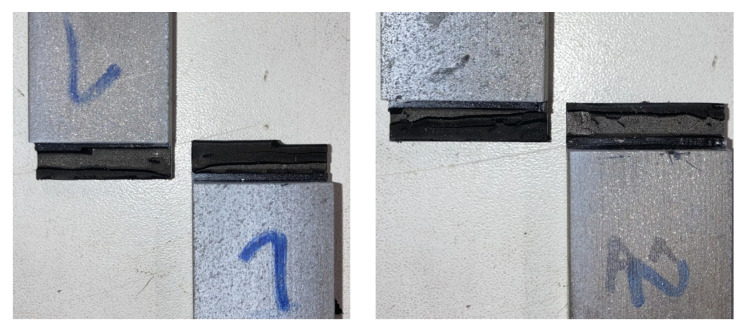
Failure mode for two TAST specimens.

**Figure 13:**
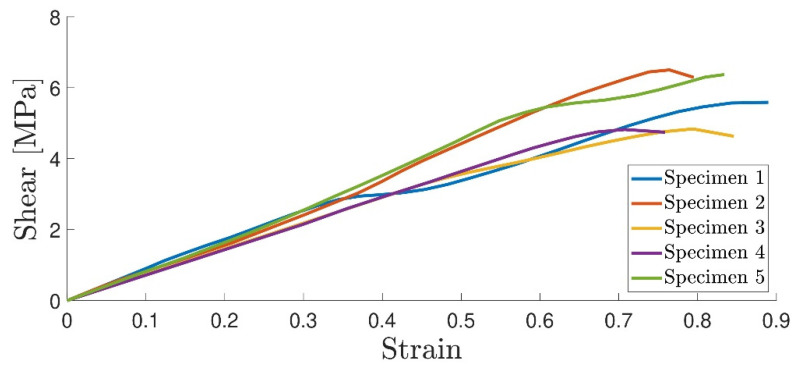
Stress–strain curve for the six TAST specimens.

The thick adherend shear test strain field was also obtained following the DIC methodology. This shows an advantage over extensometer-based displacement measurements, where both the adhesive and substrate displacements are measured [33]. The DIC allows for the characterization of the specimen displacement field. All specimens showed a similar failure mode, as illustrated in Figure 12. The stress–strain curve is displayed in Figure 13 where the shear modulus was calculated using the curve slope in the elastic regime, as well as the strength data. These results are summarized in Table 3.

By considering the Poisson’s ratio relation between the Young’s and shear modulus in isotropic materials, the shear modulus was be determined from the DIC analysis performed in the tensile test, giving a shear modulus value of the adhesive of *G* = 3.59 [MPa]. This value did not correspond to the value measured in the DIC analysis for the TAST. Should the Poisson’s ratio be calculated under the experimentally determined Young’s and shear modulus, it would lead to a value of *ν* = −0.281, which is unrealistic [42]. The shear modulus was obtained using the DIC correlation and extensometer methods, with both showing good agreement. These two methodologies were used in different specimens’ faces, since they could rotate sideways when using ductile adhesives [32]. Given the existence of layer constraints during the test, it was hard to test the material under pure shear loading. Hence, it was believed that the Poisson’s ratio was more accurately determined from the DIC method carried out in the tensile tests. Therefore, the TAST was only used to provide the maximum shear strength.

(7) $$E=2G\left(1+\nu \right)$$

#### 4.1.3. DCB Test

**Figure 14:**
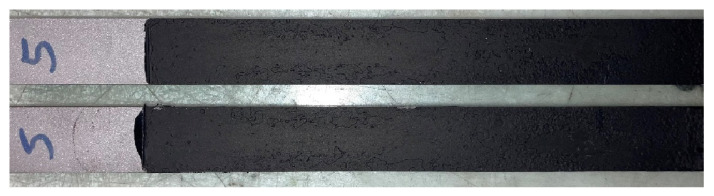
Typical fracture failure mode.

**Figure 15:**
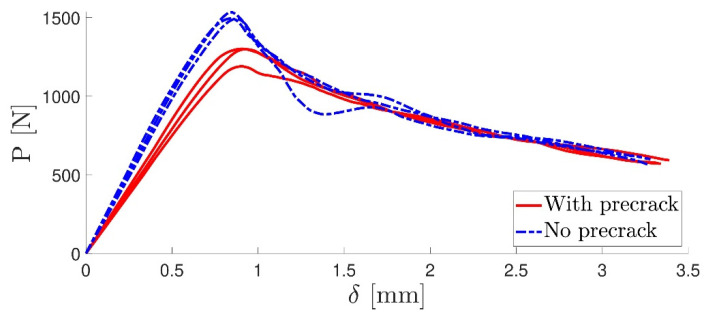
P-*δ* curve.

**Figure 16:**
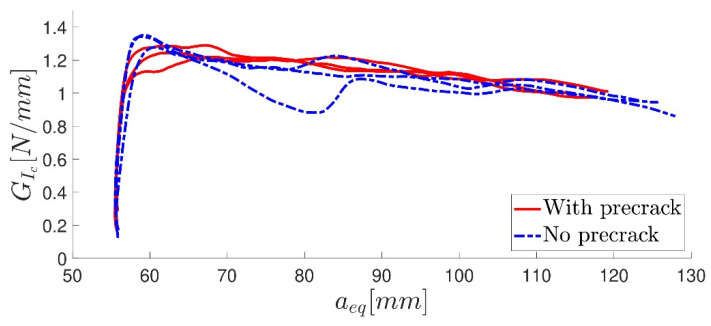
R-curve.

Usually, before fracture testing was carried out, a precrack propagation was initiated in each DCB specimen. This was performed by mounting the specimen in the quasi-static machine and applying an opening load at the same test rate. When the P-*δ* curve reached a peak, the crack had theoretically begun to propagate, and the test was stopped. Adhesives with an elastomeric behaviour possess a considerably large FPZ, and such a precracking procedure could induce multiple cracks in the opening area, originating multiple energy dissipation mechanisms [43] or damaging the initial FPZ. Given the unknown values of the critical energy release rate *G_I__c_* for the adhesive, precracking was performed in three of the six specimens, whereas the other three were not subjected to the precracking procedure. Figure 14 shows the typical DCB fracture surface for the quasi-static rate. The P-*δ* curves for the three specimens with and without the precracking procedure are displayed in Figure 15. The critical energy release rate in mode I using the CBBM [21], for both types of specimens, is shown in Figure 16. A fracture toughness value of *G_I__c_* = 1.191 ± 0.055 was found, with no significant difference between the specimens with precracking and those without.

#### 4.1.4. Mixed-Mode Test

**Figure 17:**
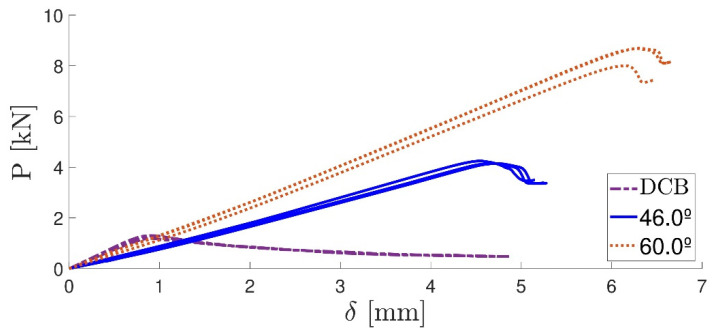
P-*δ* curves for mixed mode at 46.0°, 60.0° and 0° (DCB).

The DCB specimens were slightly opened in order to take out both the blade and calibrated tape, without compromising the initial crack and FPZ. The mixed-mode apparatus [39] was placed in the quasi-static machine and assembled by adjusting the geometrical parameters according to Table 1. The P-*δ* curves for the three best specimens (out of the six tested) were then retrieved from the two analysed angle values (Figure 17).

**Figure 18:**
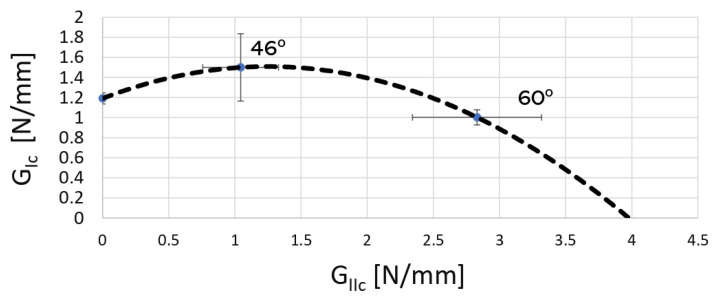
2k SPUR fracture envelope.

Table 4 summarizes the values of the critical energy release rate for mode I and II for the two studied phase angles. Figure 18 displays the extrapolated fracture envelope of the two-component adhesive, from which an estimated value of the critical energy release rate in mode II for the adhesive was found to be *G_II__c_* ≈ 4 [N/mm]. Lastly, the adhesive quasi-static mechanical properties are summarized in Table 5.

### 4.2. Influence in the Use of Additives

Three types of resin modifier additives were mixed with the 2k adhesive mixtures. Bulk and TAST procedures were carried out to compare both the tensile and shear properties to the original unmodified SPUR adhesive. Table 6 summarizes the tensile properties: young’s modulus, maximum tensile strength and maximum strain to failure.

**Figure 19:**
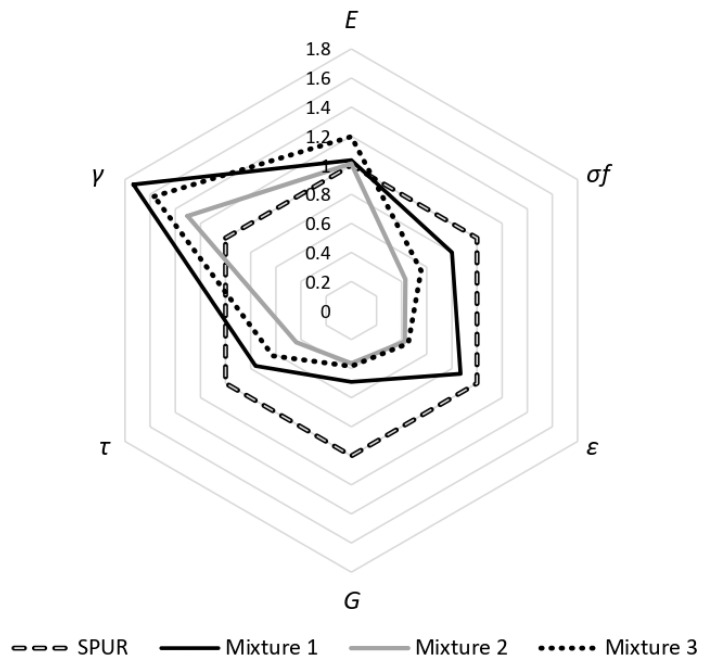
Comparison between tensile and shear properties of the normalized added adhesive properties to the 2k SPUR.

The TAST results for the different additives are shown in Table 7. Both tensile and shear properties were normalized using the properties of the unmodified 2k SPUR adhesive (Figure 19).

Table 8 summarizes the shear modulus values, estimated using the Poisson’s ratio determined for the unmodified SPUR adhesive.

### 4.3. Influence of SPUR Viscosity in G_Ic_ Value

**Figure 20:**
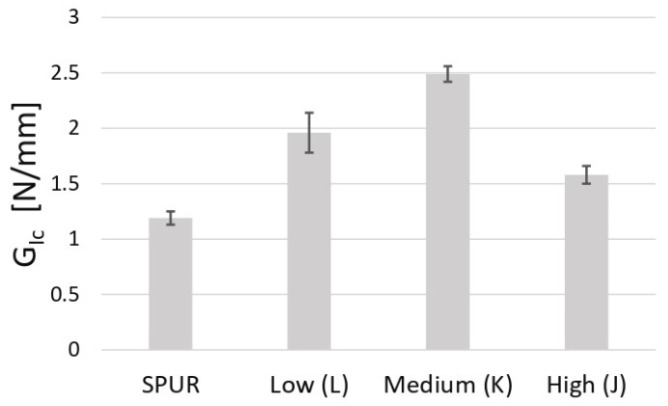
Influence of SPUR viscosity level on the *G_Ic_* value.

DCB tests were performed for the three adhesives prepared with different SPUR viscosities: low, intermediate and high. The R-curves were computed using the CBBM [34] formulation. All studied DCB specimens had cohesive failure, similar to the 2k SPUR adhesive batch characterized in the previous section. The critical energy release rate in mode I values are summarized in Table 9, and the results are compared in Figure 20.

## 5. Discussion

The data collection from other STPs and flexible adhesives indicated that the dual cure silylated polyurethane prototype adhesive shown in this study remained at the low end of the semi-structural adhesive range. The critical energy release rate values were comparable to ones for typical polyurethane adhesives and were found to be lower than standard commercial products [44,45]. This may be due to the epoxy, since epoxy adhesives possess a lower critical energy release rate when compared to PU adhesives [2]. The tensile and shear properties were still higher than those reported in the literature for silicone-based adhesives [46,47] used in the transportation industry, albeit the same could not be concluded for commonly used 2k polyurethane solutions. The SPUR adhesive does not improve both tensile and shear properties when combined with different additives. The critical energy release rate in mode I of the material shows the dependency of the SPUR level of viscosity.

## 6. Conclusions

In this paper, the mechanical properties of a new two-component dual cure SPUR adhesive were characterized at room temperature under quasi-static conditions.

- By performing tensile bulk analysis, a Young’s modulus of 10.17 [MPa] and a tensile strength of 4.16 [MPa] were determined, with a failure tensile strain of 41%. The Poisson’s ratio was determined using the DIC analysis and was found to be *ν* = 0.418.
- The TAST was performed to analyse the adhesive shear properties. The 2k formulation showed a shear vs. tensile strength ratio of 1.4. The estimated shear modulus was 3.59 [MPa] and *γ_f_* was 85%.
- Fracture tests were performed and the fracture envelope for the 2k SPUR generated. The mode I fracture toughness *G_I__c_* was found to be 1.19 [N/mm] and the estimated mode II fracture toughness *G_II__c_* was found to be 4 [N/mm]. The ratio of *G_II__c_* to *G_I__c_*, of around 3.3, was consistent with the fracture toughness ratio of other polyurethane adhesives [44].

Tensile and shear tests were conducted to analyse the behaviour of the adhesive modified with three different additives. The tensile properties with the additives were inferior in strength and elongation compared to the properties of the unmodified adhesive. The shear properties of the adhesive with the additives were also lower, but the strain to failure was doubled, reaching 140% with additives.

The study showed that the fracture toughness value in mode I depended significantly on the viscosity of the SPUR resin used in the adhesive. The adhesive with the medium viscosity SPUR exhibited the highest value of fracture toughness, with a 50% increase compared to the low viscosity SPUR adhesive.

The data collection report of other similar adhesives concluded that the prototype adhesive provides in general lower mechanical properties compared to commercial solutions used in the automotive industry.

## Figures and Tables

**Table 1:** Geometrical parameter values for each phase angle tested.

*Φ* (^0^)	*s*_1_ [mm]	*s*_2_ [mm]	*s*_3_ [mm]	*s*_4_ [mm]	*L*_1_ [mm]	2*L* [mm]
46.0	40	100	140	40	185	230
60.0	60	60	120	40	165	220

**Table 2:** Elastic data properties.

Young’s Modulus, *E*	Tensile Strength, *σ_f_*	Tensile Strain, *ε_f_*
[MPa]	[MPa]	[%]
10.17 ± 0.96	4.16 ± 0.21	0.411 ± 0.058

**Table 3:** Shear data properties.

Shear Modulus, *G*	Shear Strength, *τ_f_*	Shear Strain, *γ_f_*
[MPa]	[MPa]	[%]
7.07 ± 1.53	5.47 ± 0.74	0.825 ± 0.045

**Table 4:** Critical energy release rate values for mixed mode.

	46.0°	60.0°
*G_Ic_* [N/mm]	1.50 ± 0.34	1.00 ± 0.08
*G_IIc_* [N/mm]	1.04 ± 0.29	2.83 ± 0.49

**Table 5:** 2k SPUR mechanical properties.

Property	Units	2k SPUR
Young’s modulus, *E*	[MPa]	10.17 ± 0.96
Poisson’s ratio, *ν*	[−]	0.418 ± 0.009
Tensile failure strength, *σ_f_*	[MPa]	4.16 ± 0.21
Tensile failure strain, *ε_f_*	[%]	41.1 ± 5.8
Shear modulus, G	[MPa]	7.07 ± 1.53
Shear failure strength, *τ_f_*	[MPa]	5.47 ± 0.74
Shear failure strain, *γ_f_*	[%]	84.7 ± 11.5
Toughness in mode I, *G_Ic_*	[N/mm]	1.191 ± 0.055
Toughness in mode II, *G_IIc_*	[N/mm]	4

**Table 6:** Tensile properties with the three different additives.

	Young’s Modulus, *E*	Tensile Strength, *σ_f_*	Tensile Strain, *ε_f_*
	[MPa]	[MPa]	[%]
Mixture 1	10.52 ± 0.50	3.33 ± 0.79	0.357 ± 0.090
Mixture 2	10.28 ± 0.55	1.79 ± 0.16	0.175 ± 0.018
Mixture 3	12.17 ± 0.35	2.30 ± 0.19	0.189 ± 0.014

**Table 7:** Shear properties with the three different additives.

	Shear Modulus, G	Shear Strength, *τ_f_*	Shear Strain, *γ_f_*
	[MPa]	[MPa]	[%]
Mixture 1	3.47 ± 0.50	4.17 ± 0.44	1.610 ± 0.143
Mixture 2	2.57 ± 0.29	2.39 ± 0.16	1.210 ± 0.132
Mixture 3	2.71 ± 0.36	3.41 ± 0.25	1.457 ± 0.107

**Table 8:** Shear modulus estimation for all mixtures.

	Mixture 1	Mixture 2	Mixture 3
Shear modulus, *G* [MPa]	3.55	3.62	4.29

**Table 9:** SPUR viscosity influence in *G_Ic_* values.

	First SPUR Batch	Low Viscosity	Intermediate Viscosity	High Viscosity
Designation	[−]	L	K	J
*G_Ic_* [N/mm]	1.191 ± 0.055	1.96 ± 0.18	2.49 ± 0.07	1.53 ± 0.08

## Data Availability

Data is contained within the article.

## References

[1] Berntsen J.F., Morin D., Clausen A.H., Langseth M. (2019). Experimental investigation and numerical modelling of the mechanical response of a semi-structural polyurethane adhesive. Int. J. Adhes. Adhes..

[2] Da Silva L.F.M., Ochsner A., Adams R.D. (2018). Handbook of Adhesion Technology.

[3] Oliveira P.R., May M., Panzera T.H., Scarpa F., Hiermaier S. (2020). Reinforced biobased adhesive for eco-friendly sandwich panels. Int. J. Adhes. Adhes..

[4] Savov V., Antov P., Zhou Y., Bekhta P. (2023). Eco-Friendly Wood Composites: Design, Characterization and Applications. Polymers.

[5] Antov P., Savov V., Neykov N. (2020). Sustainable bio-based adhesives for eco-friendly wood composites. A review. Wood Res..

[6] Martinsen K., Hu S.J., Carlson B.E. (2015). Joining of dissimilar materials. Cirp Ann..

[7] Nagarajan B.M., Manoharan M. (2023). Assessment of dissimilar joining between metal and polymer hybrid structure with different joining processes. J. Thermoplast. Compos. Mater..

[8] Chen Y., Yang X., Li M., Wei K., Li S. (2019). Mechanical behavior and progressive failure analysis of riveted, bonded and hybrid joints with CFRP-aluminum dissimilar materials. Thin-Walled Struct..

[9] Pethrick R.A. (2015). Design and ageing of adhesives for structural adhesive bonding—A review. Proc. Inst. Mech. Eng. Part L J. Mater. Des. Appl..

[10] Real J.D., De Santayana M.C., Abenojar J., Martinez M.A. (2006). Adhesive bonding of aluminium with structural acrylic adhesives: Durability in wet environments. J. Adhes. Sci. Technol..

[11] Xu S., Dillard D.A., Dillard J.G. (2003). Environmental aging effects on the durability of electrically conductive adhesive joints. Int. J. Adhes. Adhes..

[12] Sousa J.M., Correia J.R., Cabral-Fonseca S. (2018). Durability of an epoxy adhesive used in civil structural applications. Constr. Build. Mater..

[13] Tong L., Sheppard A., Kelly D., Chalkley P. (1998). Effect of joint flexibility in adhesively bonded composite panel-to-flange joints. Compos. Part B Eng..

[14] Bahattab M.A., Donate-Robles J., García-Pacios V., Martín-Martínez J.M. (2011). Characterization of polyurethane adhesives containing nanosilicas of different particle size. Int. J. Adhes. Adhes..

[15] Gadhave R.V., Mahanwar P.A., Gadekar P.T. (2017). Bio-renewable sources for synthesis of eco-friendly polyurethane adhesives. Open J. Polym. Chem..

[16] Banea M.D., da Silva L.F.M. (2009). Mechanical characterization of flexible adhesives. J. Adhes..

[17] Duncan B.C., Crocker L.E. (2023). Characterisation of Flexible Adhesives for Design.

[18] Moriga T., Aoyama N., Tanaka K. (2015). Development of a polyurethane sealing gasket with excellent sealing and opening properties. Polym. J..

[19] Erbil A.E., Parlar Z., Temiz V. (2007). Performance characteristics of polyurethane foam gaskets. Arch. Mater. Sci..

[20] Qin C.L., Cai W.M., Cai J., Tang D.Y., Zhang J.S., Qin M. (2004). Damping properties and morphology of polyurethane/vinyl ester resin interpenetrating polymer network. Mater. Chem. Phys..

[21] Nakamura M., Aoki Y., Enna G., Oguro K., Wada H. (2015). Polyurethane damping material. J. Elastomers Plast..

[22] Çetin M.E. (2021). The effect of carbon nanotubes modified polyurethane adhesive on the impact behavior of sandwich structures. Polym. Compos..

[23] Gadhave R., Gadhave C., Dhawale P. (2021). Silane terminated prepolymers: An alternative to silicones and polyurethanes. Open J. Polym. Chem..

[24] Briddell B.J., Wang X., Kubish S.D. (2002). Silylated Polyurethanes for Adhesives and Sealants with Improved Mechanical Properties. U.S. Patent.

[25] Briers D., Klein J., Baetzgen R., Damke J.-E. (2019). Silylated Polyurethanes, Their Preparation and Use. U.S. Patent.

[26] Xi X., Pizzi A., Delmotte L. (2018). Isocyanate-free polyurethane coatings and adhesives from mono- and di-saccharides. Polymers.

[27] Zander L., Peng J. (2018). New silane-terminated polymers for sealants and adhesives. Adhes. Adhes. Sealants.

[28] Huber M.P., Kelch S., Berke H. (2016). FTIR investigations on hydrolysis and condensation reactions of alkoxysilane terminated polymers for use in adhesives and sealants. Int. J. Adhes. Adhes..

[29] Cao C.L., Cheng J., Liu X.D., Wang R., Zhang J.Y., Qu J., Jaeger U. (2012). Study of properties of one-component moisture-curable polyurethane and silane modified polyurethane adhesives. J. Adhes. Sci. Technol..

[30] Devroey D.R.E., Homma M. (2001). Blends of silyl-terminated polyethers and epoxides as elastic adhesives. Int. J. Adhes. Adhes..

[31] Bitenieks J., Meri R.M., Zicans J., Berzins R., Umbraško J., Rekners U. (2016). Modified silyl-terminated polyether polymer blends with bisphenol a diglycidyl ether epoxy for adhesive applications. IOP Conf. Ser. Mater. Sci. Eng..

[32] Da Silva L.F.M., Dillard D.A., Blackman B.R.K., Adams R.D. (2012). Testing Adhesive Joints—Best Practices.

[33] Banea M., Silva L.F.M., Campilho R. (2011). Mechanical characterization of a high temperature epoxy adhesive. Weld. Equip. Technol..

[34] (1988). Méthode de Préparation de Plaques D’adhésifs Structuraux Pour la Réalisation D’éprouvettes D’essai de Caractérisation.

[35] (1993). Adhesives—Determination of Shear Behaviour of Structural Bonds, Part 2: Thick-Adherend Tensile-Test Method.

[36] (2009). Adhesives—Determination of the Mode 1 Adhesive Fracture Energy of Structural Adhesive Joints Using Double Cantilever Beam and Tapered Double Cantilever Beam Specimens.

[37] De Moura M.F.S.F., Campilho R.D.S.G., Gonçalves J.P.M. (2008). Crack equivalent concept applied to the fracture characterization of bonded joints under pure mode i loading. Compos. Sci. Technol..

[38] Reis M.D., Banea M., Silva L.F.M., Carbas R. (2019). Mechanical characterization of a modern epoxy adhesive for automotive industry. J. Braz. Soc. Mech. Sci. Eng..

[39] Costa M., Carbas R., Marques E., Viana G., da Silva L.F.M. (2017). An apparatus for mixed-mode fracture characterization of adhesive joints. Theor. Appl. Fract. Mech..

[40] Owens A.T., Tippur H.V. (2021). Measurement of mixed-mode fracture characteristics of an epoxy-based adhesive using a hybrid digital image correlation (DIC) and finite elements (FE) approach. Opt. Lasers Eng..

[41] De Moura M.F.S.F., Campilho R.D.S.G., Gonçalves J.P.M. (2009). Pure mode ii fracture characterization of composite bonded joints. Int. J. Solids Struct..

[42] Karpiesiuk J. (2020). Young’s modulus and poisson’s ratio of polyurethane adhesive in lightweight floor system. Mod. Approaches Mater. Sci..

[43] Saleh M.N., Budzik M.K., Saeedifar M., Zarouchas D., De Freitas S.T. (2022). On the influence of the adhesive and the adherend ductility on mode i fracture characterization of thick adhesively-bonded joints. Int. J. Adhes. Adhes..

[44] Ribas M.J.P., Akhavan-Safar A., Pigray N., Carbas R.J.C., Marques E.A.S., Borges C.S.P., Wenig S., da Silva L.F.M. (2023). From high strain rates to elevated temperatures: Investigating mixed-mode fracture behaviour in a polyurethane adhesive. Polymers.

[45] Banea M.D., da Silva L.F.M., Campilho R.D.S.G. (2015). The effect of adhesive thickness on the mechanical behavior of a structural polyurethane adhesive. J. Adhes..

[46] Yasmina B., Sami N., Salah M., Lucas F.M., Mohamed H., Moez B.S.A. (2016). Effect of adhesive thickness and surface roughness on the shear strength of aluminium one-component polyurethane adhesive singlelap joints for automotive applications. J. Adhes. Sci. Technol..

[47] Machado J.J.M., Gamarra P.M.-R., Marques E.A.S., da Silva L.F.M. (2018). Numerical study of the behaviour of composite mixed adhesive joints under impact strength for the automotive industry. Compos. Struct..

